# Supplementary material for: Anti-Inflammatory Effects of Honeysuckle Leaf Against Lipopolysaccharide-Induced Neuroinflammation on BV2 Microglia
Source: Nutrients. 2024 Nov 19;16(22):3954. doi: 10.3390/nu16223954 (PMC11597670; doi:10.3390/nu16223954)
Supplement: Supplementary file 1 [file nutrients-16-03954-s001.zip › nutrients-3222368-supplementary.pdf]

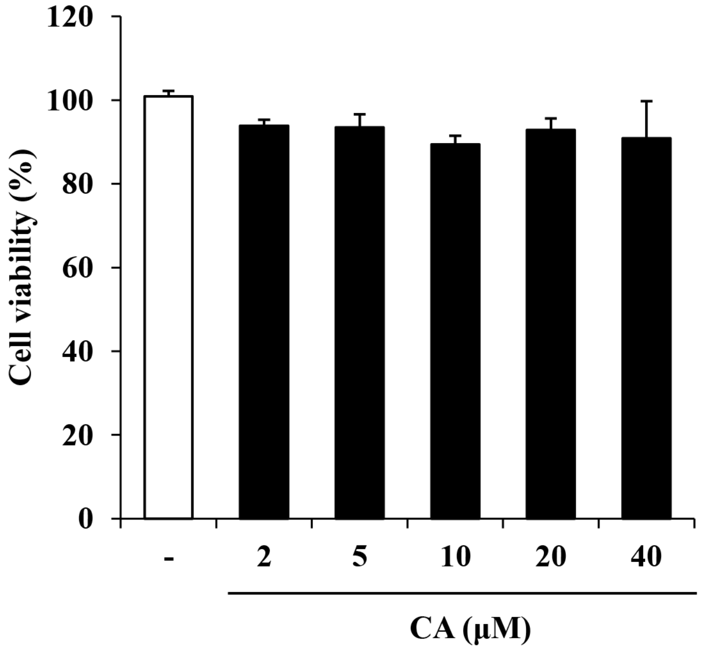

Figure S1. The cytotoxicity of the chlorogenic acid (CA) in BV2 microglial cells. Cells were cultured with CA at indicated concentrations and after 24 h, cell viability was assessed using the 3-(4,5-dimethylthiazol-2-yl)-2,5 diphenyl tetrazolium bromide (MTT) reagent. Data are expressed as means ± standard error of the mean (SEM). Results are representative of three experiments.

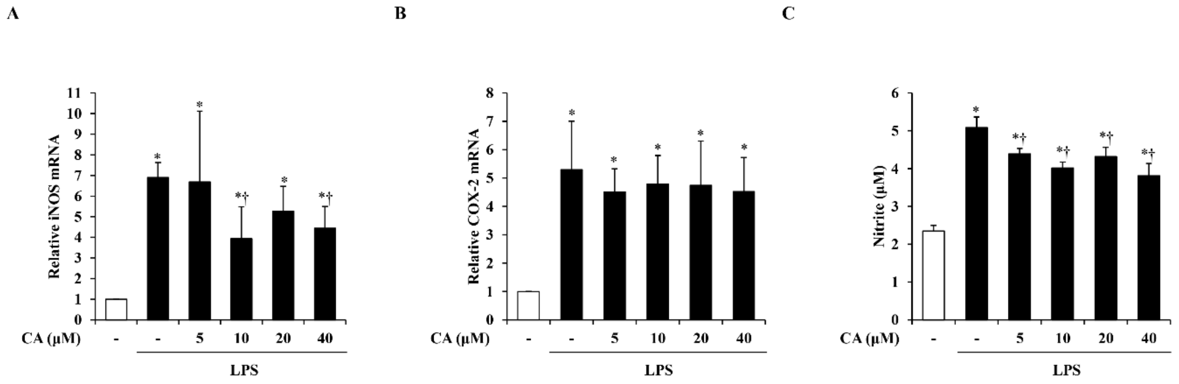

Figure S2. Effect of chlorogenic acid (CA) on the production of inflammatory mediators in BV2 microglial cells activated by lipopolysaccharide (LPS). BV2 cells were pre-treated with CA at indicated concentrations for 1 h and next co-incubated with LPS (1 μg/mL) for 6 h. Afterward, mRNA levels of (A) inducible nitric oxide synthase (iNOS) and (B) cyclooxygenase (COX)-2 were assessed using real-time reverse transcription-polymerase chain reaction (RT-PCR). (C) The cells were pre-treated with CA and co-incubated with LPS (1 μg/mL) for 24 h. Next, nitrite production was evaluated by the Griess reaction. Data are expressed as means ± standard error of the mean (SEM). Results are representative of three experiments. *$p < 0.05$ vs. DMSO treatment alone; †$p < 0.05$ vs. LPS treatment alone.

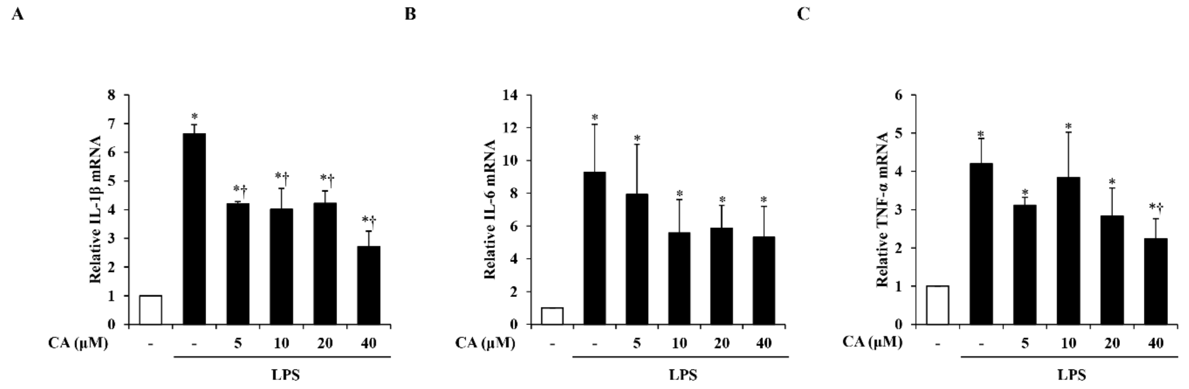

Figure S3. Effect of chlorogenic acid (CA) on the secretion of inflammatory cytokines in BV2 microglial cells. The cells were pre-treated with CA at indicated concentrations for 1 h and next co-incubated with lipopolysaccharide (LPS) (1 μg/mL) for 6 h. The mRNA levels of (A) interleukin (IL)-1β, (B) IL-6, and (C) tumor necrosis factor (TNF)-α were assessed using real-time reverse transcription-polymerase chain reaction (RT-PCR). Data are expressed as means ± standard error of the mean (SEM). Results are representative of three experiments. **p* < 0.05 vs. DMSO treatment alone; †*p* < 0.05 vs. LPS treatment alone.
